# Supplementary material for: Immunoproteasome Activity and Content Determine Hematopoietic Cell Sensitivity to ONX-0914 and to the Infection of Cells with Lentiviruses
Source: Cells. 2021 May 12;10(5):1185. doi: 10.3390/cells10051185 (PMC8150886; doi:10.3390/cells10051185)
Supplement: Supplementary file 1 [file cells-10-01185-s001.zip › cells-1212761-supplementary.pdf]

Supplementary Methods

Cloning of cells and synchronization of cellular clones

Cells were cloned by limiting dilution. After the expansion, obtained clones were stored in liquid nitrogen. For synchronization 2×10^5 cells were washed with FBS-medium (2×) and incubated in FBS-RPMI for 24 hours. After that FBS was added to the cell culture medium.

Molecular cloning and plasmid purification

The total mRNA and cDNA were obtained from U937 cells as described above. Then the fragments corresponding to the proteasome subunit genes *PSMB8*, *9*, *10* were amplified by PCR with primers listed in Table S1 and cloned into the commercial expression vector pcDNA3.1- (Invitrogen, Carlsbad, CA, USA). The plasmids were amplified in *E.coli* and purified using Qiagen plasmid mini kit (Qiagen, Hilden, Germany) according to manufacturer's instructions. To increase transfection efficacy and reduce putative side effects plasmids were purified from endotoxin using MiraCLEAN kit (Mirus bio, Madison, WA, USA).

Transfection, preparation of lysates and Western blotting

The HEK 293 cells were transfected with obtained plasmids using Mirus TrasIT 293 (MirusBio, Madison, WA, USA) transfection reagent according to manufacturer's instructions. Forty-eight hours post transfection cells were lysed as described in the M&M section of the main text. Western blots were performed with either rabbit anti-β1i (Abcam, Cambridge, UK), or rabbit anti-β2i (Abcam, Cambridge, UK), or rabbit anti-β5i (Abcam, Cambridge, UK) primary antibodies and goat anti-rabbit HRP conjugates (Abcam, Cambridge, UK). Blots were revealed as described in the M&M section of the manuscript.

Supplementary Figure

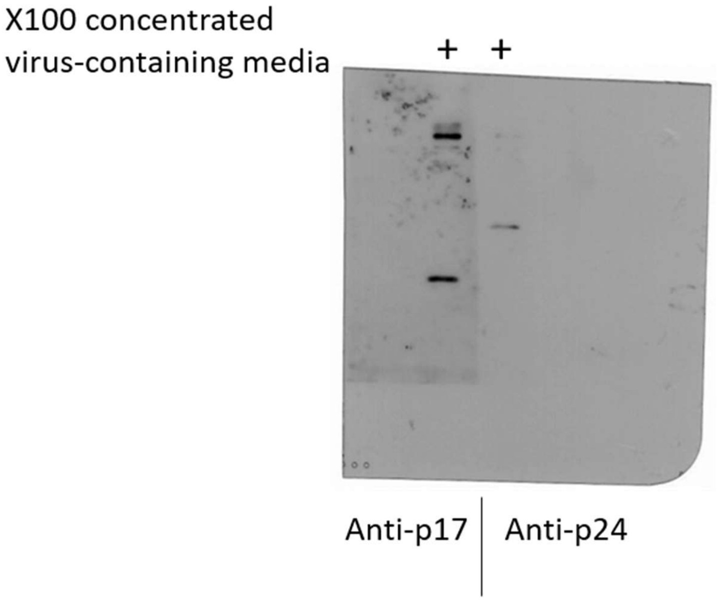

Figure S1. Western blotting of 100 times concentrated virus-containing media with antibodies to the HIV-1 capsid proteins, original image. Anti-p17 (left part) and anti-p24 (right part). Uncropped image, corresponding to Figure 4a in the main text.

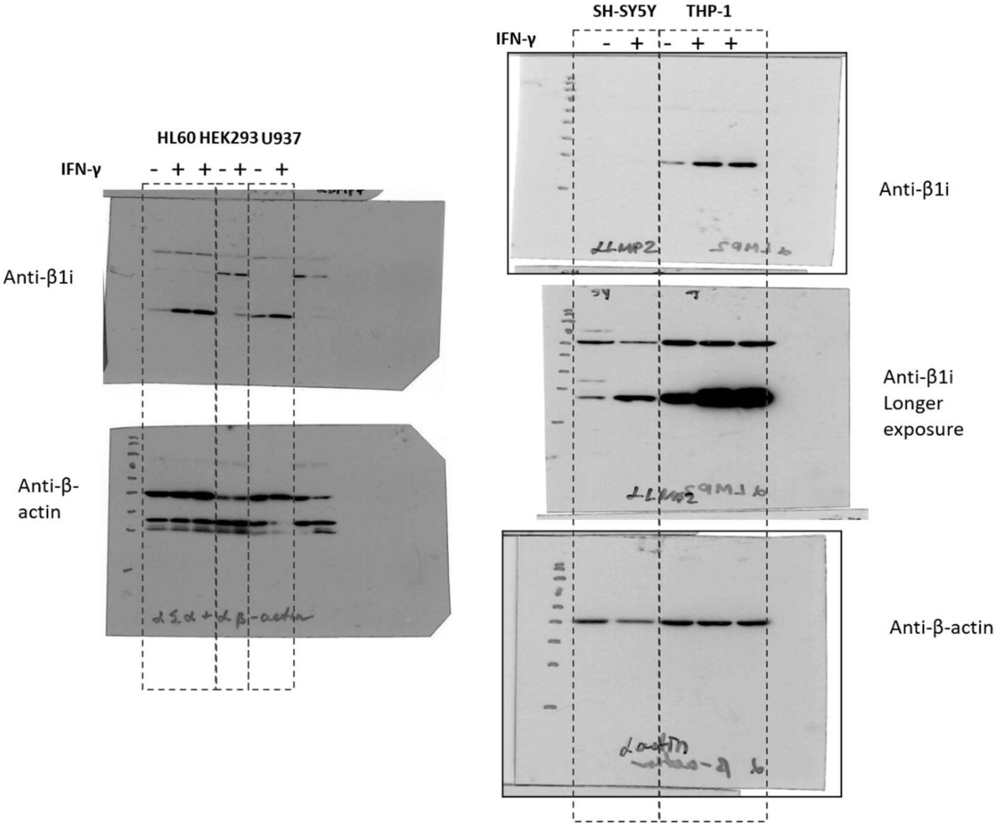

Figure S2. Levels of immunoproteasome subunit β1i in IFN-γ-treated cells, original images. The HL-60, THP-1, U937, SH-SY5Y and HEK293 cells were treated with 1000 U/ml of recombinant human IFN-γ for 48 hours. After that cells were lysed and the β1i levels in lysates were determined by Western blotting. Areas with specific samples are enclosed in squares. Uncropped images, corresponding to Figure 5b in the main text.

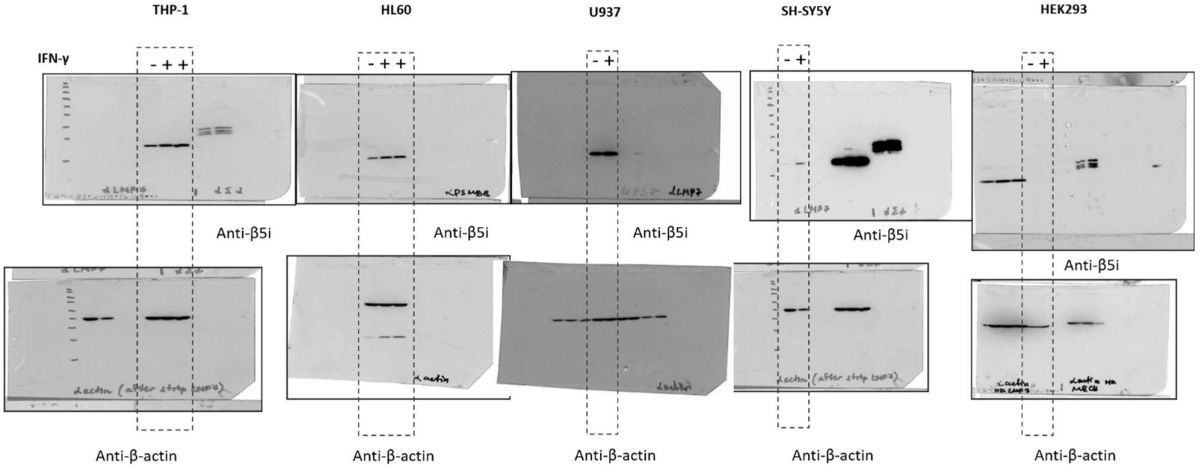

Figure S3. Levels of immunoproteasome subunit β5i in IFN-γ-treated cells, original images. The HL-60, THP-1, U937, SH-SY5Y and HEK293 cells were treated with 1000 U/ml of recombinant human IFN-γ for 48 hours. After that cells were lysed and the β5i levels in lysates were determined by Western blotting. Areas with specific samples are enclosed in squares. Uncropped images, corresponding to Figure 5b in the main text.

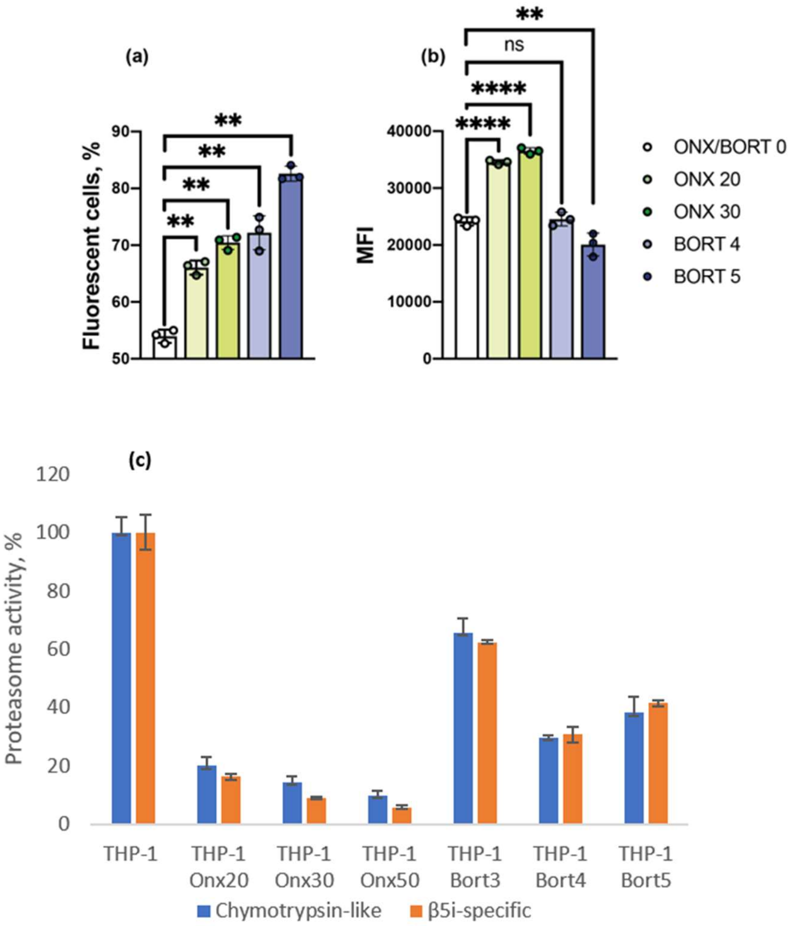

Figure S4. Lentiviral transduction of THP-1 cells following treatment with different concentrations of ONX-0914 or Bortezomib. THP-1 cells were incubated with 20 and 30 nM of ONX-0914 or 4 and 5 nM of Bortezomib for 6 hours prior to the addition of lentiviral particles. After administration of viruses cells were incubated for additional 72 hours. Cells were collected and per cent of fluorescent cells (a) and mean fluorescence intensity (b) were measured using LSRFortessa flow cytometer (BD Biosciences). Dots represent individual replicates. Bars represent standard deviation. Three independent repeats were performed. Two-way ANOVA test for multiple comparisons with no correction was used. Asterisks: * - p-value <0.1; ** - p-value <0.01; *** - p-value < 0.001; **** - p-value <0,0001; ns – not significant. (c) Chymotrypsin-like and β5i-specific activities in THP-1 cells after incubation with proteasome inhibitors. Tests were performed in triplicates. Bars represent standard deviation.

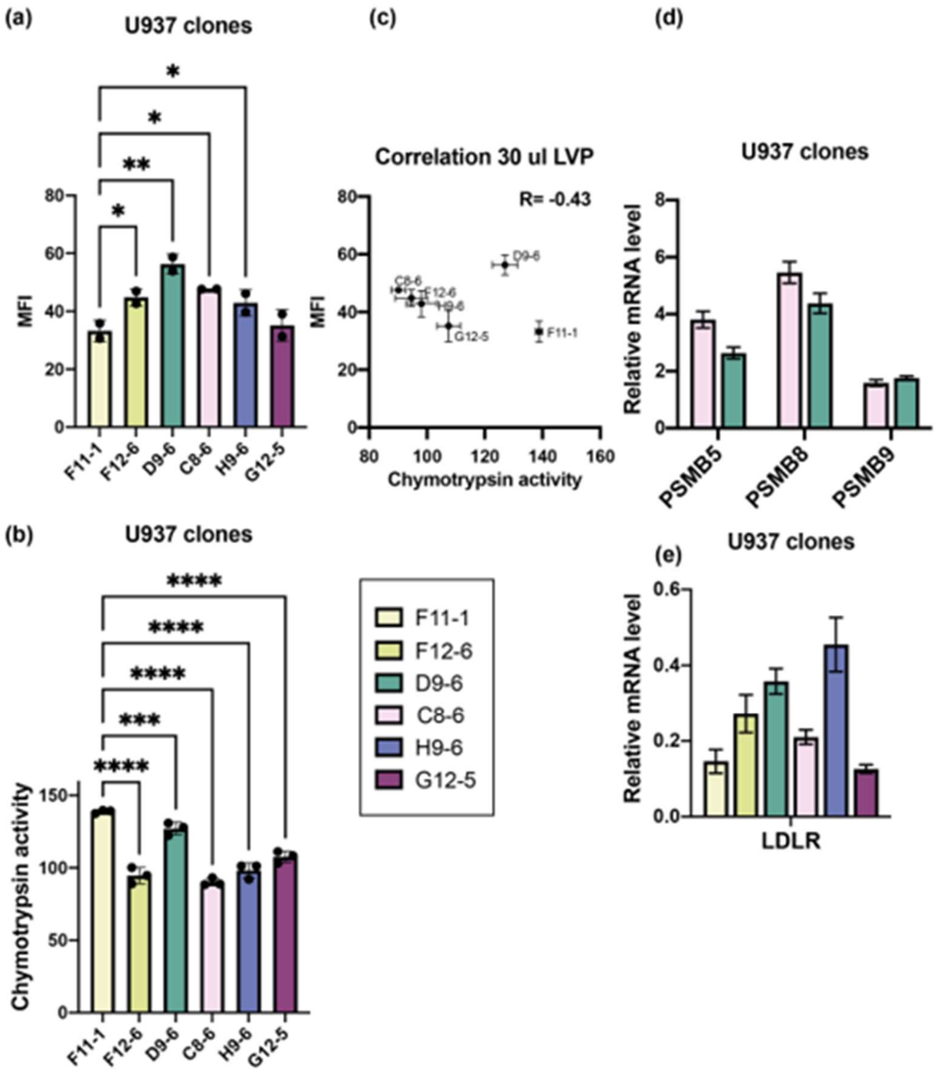

Figure S5. Correlation of transduction efficacy and proteasome activity in U937 cell line clones. (a) Transduction efficacy of the clones after treatment with lentiviral particle (LVP)-containing media. (b) Chymotrypsin-like activity in the clones. Proteasome activity was determined in cellular lysates using Suc-LLVY-AMC fluorogenic substrate. (c) Estimation of the correlation between transduction efficacy and proteasome activity in U937 cell line clones treated with LVP-containing media. Non-parametric Spearman correlation was calculated using GraphPad 9.1. Correlation coefficient (R) is shown on the graph. (d) The *PSMB5, PSMB8, PSMB9* mRNA expression levels in U937 clones C8-6 and C9-6. Bars represent standard deviation. Tests were performed in triplicates. (e) The LDLR expression in F11-1, D9-6 and C8-6 clones. Three technical repeats were performed for each sample. Multiple comparisons were performed using one-way ANOVA and false discovery rate corrections. Asterisks: *- p-value < 0.05, ** - p-value < 0.01, *** - p-value < 0.001, **** - p-value < 0.0001.

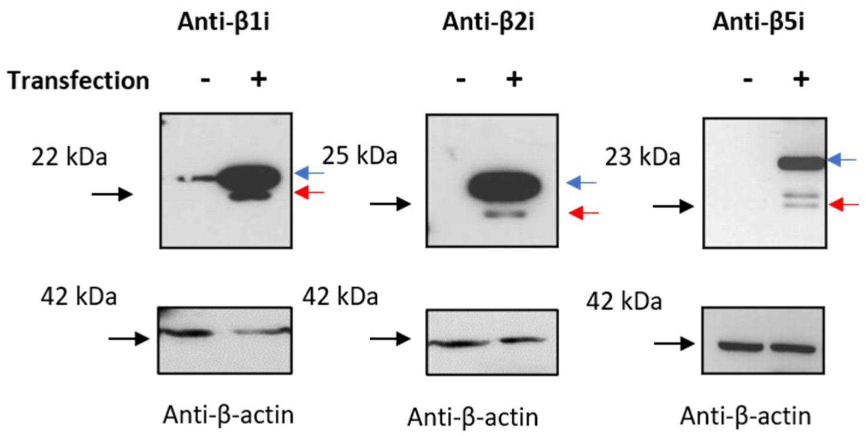

Figure S6. Expression of recombinant immunoproteasome subunits in transfected HEK 293 cells. HEK 293 cells were transfected with plasmids, encoding human β1i, β2i and β5i. Forty-eight hours post-transfection cells were lysed, and the presence of specific proteins was assessed by Western blotting. Blue arrows indicate precursor proteins, red arrows indicate cleaved subunits.

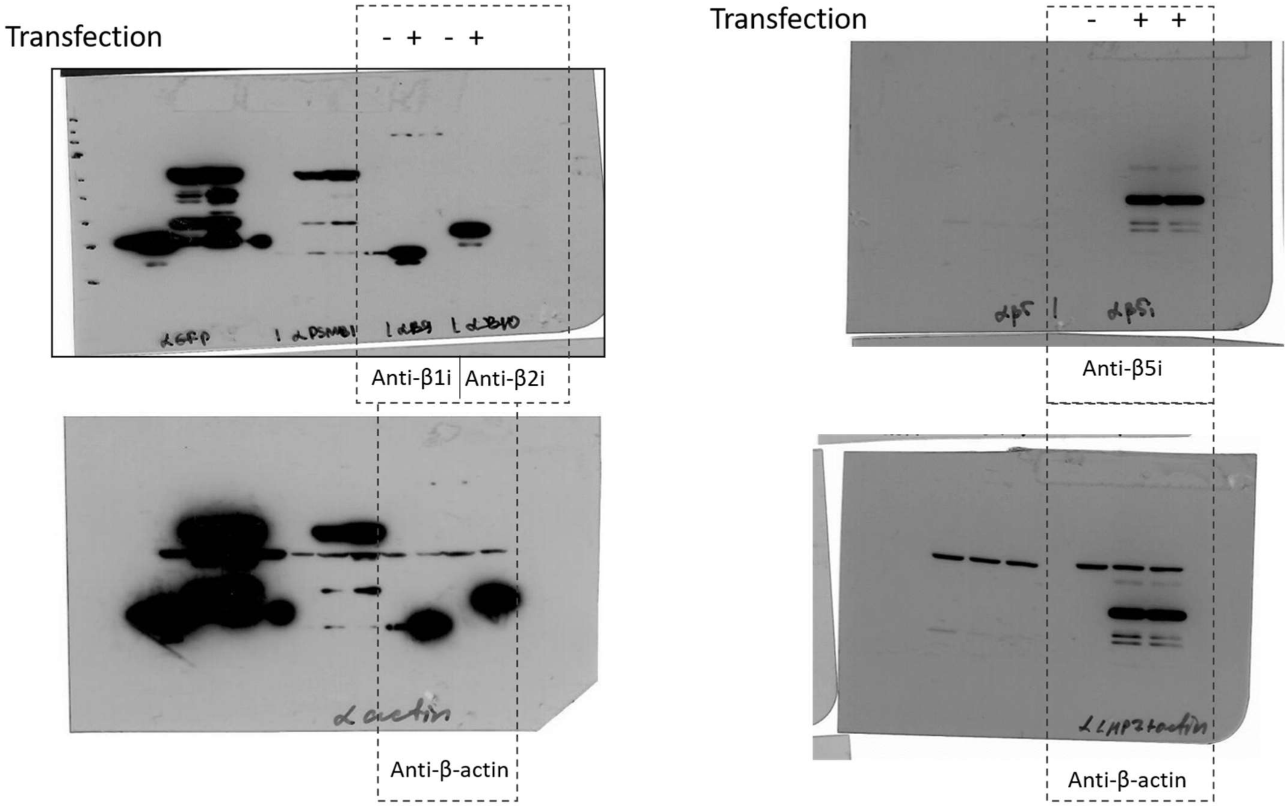

Figure S7. Expression of recombinant immunoproteasome subunits in transfected HEK 293 cells, original images. HEK 293 cells were transfected with plasmids, encoding human β1i, β2i and β5i. Forty-eight hours pos-transfection cells were lysed, and the presence of specific proteins was assessed by the Western blotting. Areas with specific samples are enclosed in squares. Uncropped images, corresponding to Figure S6.

Table S1. Primers used in the study.

Primer	Sequence 5'→3'
PSMB5 fw	CTCCAAACTGCTTGCCAAC
PSMB5 rev	GTTCCCTTCACTGTCCACG
PSMB6 fw	AAGCCGAGAAGTTTCCACT
PSMB6 rev	GCGATGTAGGACCCAGT
PSMB7 fw	CTGAAGGGATGGTTGTTG
PSMB7 rev	CAGGTTGGAAGAAATGAGC
PSMB8 fw	GGTGAACAAGGTGATTGAG
PSMB8 rev	GTTCTCCATTTCGCAGATAG
PSMB9 fw	GCTGCTGATGCCCAAGC
PSMB9 rev	GCTGATATTTCTCACCACATTTGC
PSMB10 fw	GGTTCCAGCCGAACATGA
PSMB10 rev	ATGCGTCCACATTGCCC
Actb fw	TTGGCAATGAGCGGTTCC
Actb rev	GAGTTGAAGGTAGTTTCGTGG
T7 Primer	TAATACGACTCACTATAGGG
PSMB8_complF	CCGAGAGCTAGCAGATCTCTGG
PSMB8_complR	CTGCCACCGGTACCATTATTG
PSMB9_complF	ACAGCTAGCGGGATGCTGCGGGCGGGAGCAC
PSMB9_complR	ACAAAGCTTTCACTCATCATAGAATTTTGG
PSMB10_complF	ACAGCTAGCAAGATGCTGAAGCCAGCCCTGGAG
PSMB10_complR	ACAAAGCTTTTACTCCACCTCCATAGCCTG
CXCL8F	CAGTTTTGCCAAGGAGTGCT
CXCL8R	GTTTTCCTTGGGGTCCAGACA
IL1b_F	GATGGCTTATTACAGTGGCAA
IL1b_R	GTCGGAGATTCGTAGCTGGA

TNFa_F	TAGCCCATGTTGTAGCAAACCC
TNFa_R	TGAGGAGCACATGGGTGGAG
IL6_F	ACTCACCTCTTCAGAACGAATTG
IL6_R	CCATCTTTGGAAGGTTCAGGTTG
